# Dual‐Stimuli Responsive and Sustainable PLA/APHA/TPU Blend for 4D Printing

**DOI:** 10.1002/marc.202500414

**Published:** 2025-08-14

**Authors:** Shafahat Ali, Mamoun Alshihabi, Logan Beard, Ibrahim Deiab, Salman Pervaiz

**Affiliations:** ^1^ Advanced Manufacturing Lab (AML) School of Engineering University of Guelph Guelph Ontario Canada; ^2^ Australian College of Kuwait Kuwait; ^3^ Department of Industrial and Mechanical Engineering Rochester Institute of Technology Dubai United Arab Emirates

**Keywords:** 4D printing, biodegradable SMP, heat‐activated shape memory polymer, orthopedic devices, recovery mechanics, soft robotics

## Abstract

4D printing of shape memory polymers (SMPs) offers transformative potential for patient‐specific medical devices, yet current SMPs often face a trade‐off between mechanical toughness and low‐temperature activation. This study presents a novel PLA/APHA/TPU blend filament for 3D printing that overcomes this limitation by combining high strength and flexibility with low‐temperature shape memory activation—features not previously achieved in PLA‐based SMPs. The uniform dispersion of TPU and APHA in the PLA matrix creates a composite with enhanced tensile strength, modulus, and elongation, addressing the brittleness typical of neat PLA. The optimized 60/20/20 wt.% formulation enables rapid shape recovery at ∼39.5°C, significantly below PLA's glass transition, with near‐complete shape fixity (∼100%) and high recovery ratios (>92%) under both thermal and mechanical stimuli. This dual‐responsive behavior is driven by the synergistic roles of TPU (providing ductility) and APHA (enhancing flexibility and thermal sensitivity). The composite also retains excellent printability and biocompatibility, making it ideal for next‐generation biomedical SMP applications such as 4D‐printed orthopedic braces, soft robotic actuators, and adaptive implants. Using bio‐based, biodegradable polymers, this work advances eco‐friendly, high‐performance SMPs for additive manufacturing, setting a new benchmark for PLA‐based 4D‐printable materials.

## Graphical abstract

This study introduces PLA/APHA/TPU shape memory composites for 4D printing, emphasizing enhanced flexibility, toughness, and thermal responsiveness. Detailed experimental methods, rheological, thermal, mechanical analyses, and shape recovery mechanisms are presented. Results demonstrate superior shape fixity, recovery performance, and biomedical applicability, positioning the composite as a promising material for adaptive medical devices and soft robotics.

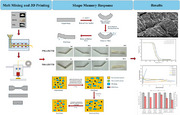

**Abbreviations**
4D: four‐dimensional
APHA: amorphous polyhydroxyalkanoates
DSC: differential scanning calorimetry
DMA: dynamic mechanical analysis
FDM: fused deposition modeling
PLA: polylactic acid
Rf: shape fixation ratio
Rr: shape recovery ratio
SEM: scanning electron microscopy
SMP: shape memory polymer
Tg: glass transition temperature
Tm: melting temperature
Tcc: cold crystallization temperature
TGA: thermogravimetric analysis
TPU: thermoplastic polyurethane

## 1 Introduction

Recently, research has focused on combining shape memory polymers (SMPs) and 3D printing technologies, resulting in a new field called 4D printing [1, 2]. SMPs are capable of reversibly regaining their original shape when subjected to external stimuli such as electricity [3], light [4], heat [5], or magnetic fields [4], which are appealing to expanding research attention. Therefore, 3D‐printed SMPs can be used to create an array of intelligent devices for various applications in fields such as electronics [6], medical robotics [7], aerospace [8], sensors [9], and biomedical [10, 11, 12, 13].

Over the past few years, a variety of SMP matrix materials have been investigated, including polylactic acid (PLA) [14, 15, 16], polyurethane (PU) [17, 18], polycaprolactone (PCL) [15, 19], poly(vinyl alcohol) (PVA) [20, 21], polyethylene terephthalate glycol (PETG) [22], and various other polymer blends [23, 24]. PLA is a semicrystalline aliphatic polyester that has high strength, modulus, and shape memory properties. The crystalline phase of the material served as a fixative phase, and the amorphous phase served as a shifting phase [25, 26]. PLA's inherent brittleness and low toughness may lead to cracking or failure due to its lower dynamic adaptability for industrial devices [27, 28]. SMPs can be developed by blending two or more thermoplastic polymers to achieve tailored shape fixity and recovery ratios.

SMP blends can overcome the disadvantages of two different matrix systems while incorporating their strengths. TPU is a block copolymer consisting of both soft and hard segments, offering outstanding elasticity and shape memory, which makes it an ideal matrix material for SMP applications [29]. In previous studies, TPU was shown to improve PLA's shape memory function and to serve as a stress concentrator, preventing irreversible damage [30]. PLA was mixed with 20 wt.% and 50 wt.% of TPU for a biomedical application using a melt mixing process. TPU with a 20 wt.% composition showed a higher degree of shape recovery [31]. PLA blend was investigated for 3D printing and found that adding PHA to PLA significantly improved its ductility [32]. Polyhydroxyalkanoates (PHA) have emerged as interesting alternatives in medical and biomedical applications [33] due to their biocompatibility and biodegradability [34, 35]. PHA is expected to overgrow in the biomedical market between 2023 and 2032, with a compound annual growth rate of 10.7% [36].

Extensive research has been conducted on PLA/TPU systems, focusing on their blend ratios, miscibility, and unique structural characteristics to enhance shape memory behavior in magnetic environments. To achieve electro‐induced shape memory effects, carbon nanotubes (CNT) and various nanoparticles have been incorporated into PLA/TPU SMP systems in multiple studies. An electro‐induced hybrid shape memory device developed by Dong et al. [37] showed improved joule heating and shape memory performance when CNTs were present in PLA/TPU systems. Zhao et al. [38] developed an effective magneto‐response SMP for biomedical applications, such as tracheal scaffolds or bone tissue scaffolds. Besides, some studies have attempted to induce thermal shape memory by introducing CNT into PLA/TPU systems.

X. Huang et al. [39] studied CNT combined with PLA/TPU to produce thermal shape memory, and this combination demonstrated a significant improvement in shape memory performance (both recovery and fixity). In the literature, various types of additives have been investigated, including graphene oxide (GO), which has improved the mechanical and thermal properties of the polymer [40]. It is particularly suitable for use in biomedical applications due to its exceptional thermal conductivity [41], biocompatibility [42, 43], and mechanical strength [40, 41]. The principal challenge with SMP systems is achieving an effective shape memory effect while delivering satisfactory mechanical capacities.

To address the existing challenge in SMP design—balancing mechanical strength with shape recovery performance at low temperatures—we developed a novel 4D‐printable SMP blend based on a carefully engineered combination of PLA, APHA, and TPU. This new blend achieves low‐temperature actuation (∼39.5°C–40°C) without sacrificing mechanical robustness, representing the first example of a PLA‐based SMP that successfully combines high mechanical strength, enhanced elasticity, and low‐temperature responsiveness. Existing PLA‐based SMPs typically suffer from high activation thresholds (∼60°C–70°C) and poor toughness, limiting their effectiveness in demanding applications such as biomedical implants, soft robotics, and adaptive aerospace components. This work overcomes these limitations by combining the complementary benefits of PLA (high strength and stiffness), TPU (elasticity and impact resistance), and APHA (biocompatibility and flexibility) to create a mechanically tough yet highly responsive SMP system. Through systematic variation of blend ratios, we optimized the material's thermal, mechanical, and morphological properties for 3D printing and evaluated its shape memory behavior under both thermal and mechanical stimuli. The optimized 60/20/20 wt.% PLA/APHA/TPU formulation demonstrated an impressive 500% increase in elongation at break—exceeding the performance of existing PLA‐based SMPs, which typically achieve less than 150% elongation. The material exhibited a rapid and efficient shape recovery rate of over 99% and nearly 100% shape fixity at body‐safe temperatures (∼40°C), ensuring consistent and reliable actuation under real‐world conditions. This combination of toughness, flexibility, and low‐temperature actuation sets a new benchmark for PLA‐based SMPs, addressing the trade‐off between stiffness and shape responsiveness observed in conventional systems.

The synergistic interaction between the polymer components produced a unique balance of strength, toughness, and thermal sensitivity, enabling the blend to sustain complex loading conditions while retaining rapid shape recovery. This high performance positions the material as an ideal candidate for 4D‐printed orthopedic braces, vascular stents, and soft robotic actuator applications where reliable shape transformation under mild thermal conditions is essential. Furthermore, enhanced energy absorption and impact resilience of the blend open new possibilities in aerospace engineering (e.g., morphing wing structures) and smart textiles (e.g., temperature‐responsive garments). Moreover, the use of biodegradable and bio‐derived components (PLA and APHA) provides an eco‐friendly alternative to petroleum‐based SMPs, ensuring excellent biocompatibility and reducing environmental impact. This work establishes a new benchmark for PLA‐based SMPs, combining low‐temperature actuation, high strength, and biodegradability, while expanding the scope of 4D printing into biomedical, aerospace, and smart textile applications.

## 2 Experimental Section

### 2.1 Materials

The 3D printing process used a PLA (type: 3D870, from Nature Works LLC, USA), APHA (GENECIS Bioindustries, Canada), and TPU (type: 93A, from filament2print Company, Spain) matrix. All of these were used directly without any further purification.

### 2.2 Preparation of Filament and Samples from PLA, APHA, and TPU

**FIGURE 1:**
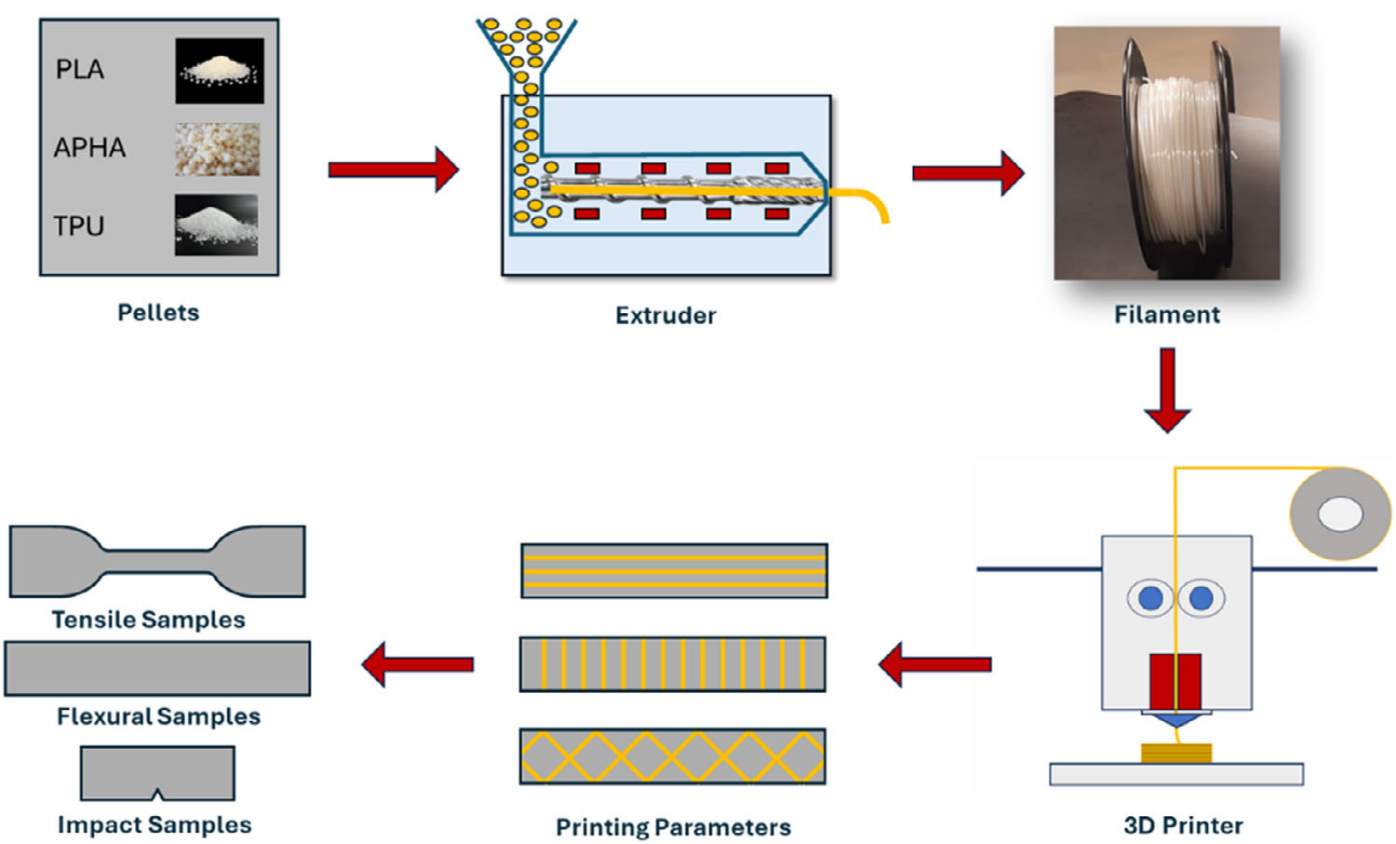
Schematic of the fabrication process for 3D‐printed samples, showing material blending, filament extrusion, and layer‐by‐layer deposition using FDM.

A filament made of PLA, APHA, and TPU is illustrated in Figure 1. The PLA, APHA, and TPU were first dried at 80°C for 3 h before being used to keep the moisture under 1% wt. A weight‐to‐volume ratio of 50/30/20, 40/40/20, and 60/20/20 was used in this study. The filament was produced using a 3Devo Composer 350 twin‐screw extruder, achieving a diameter of 1.75 ± 0.07 mm. The extruder's screw rotation speed was set to 4.3 rpm, and the fan speed was maintained at 100%. The temperature settings for the four zones of the extruder, from the feeding position to the die, were as follows: 170°C, 185°C, 180°C, and 170°C. The initial zone temperature was kept lower to prevent viscous flow problems that might obstruct the feeding port. In contrast, the following melting and homogenization zones operated at elevated temperatures, ensuring thorough polymer melting and uniform mixing. Three different filaments comprised of PLA/APHA/TPU Blend were prepared. PLA/PHA/TPU filaments with different Percentages were described as P60/A20/T20, P50/A30/T20, and P40/A40/T20.

The PLA/PHA/TPU specimens were produced using a Bambu P1S FDM 3D printer (Shanghai, China). The specimen designs were created in SolidWorks and subsequently transferred to Bambu Studio for slicing in STL format. Prior to 3D printing, the filaments underwent drying in an oven at 50°C for 24 h to eliminate moisture. The specimen preparation parameters included a nozzle diameter of 0.8 mm, an infill density of 100%, a bed temperature of 50°C, and a rectilinear infill pattern. Samples designated for tensile, flexural, and impact testing were fabricated and assessed following ASTM D638 Type IV, ASTM D790, and ASTM D256 standards.

#### 2.2.1 Design of Experiment

Taguchi's method enables the determination of optimal conditions with a minimal number of experiments, thereby significantly reducing time and costs. This approach utilizes various orthogonal arrays as experimental matrices, based on the number of parameters and their levels. To evaluate the impact of layer height, nozzle temperature, and raster angle on the tensile, flexural, and impact properties of the printed material, a Taguchi design of experiments was employed. Layer height was chosen to be between 25% and 75% of the nozzle diameter, following established guidelines. The nozzle temperature was set based on preliminary experiments. Raster angles were varied to examine their influence on surface finish, mechanical properties, and print quality. An L9(3^3) orthogonal array was used to represent the three factors, each at three levels, as detailed in Tables 1 and 2. Each condition was tested three times to ensure reliable results. The selected levels and experimental matrices allowed for the systematic variation of input variables, enabling the identification of optimal conditions.

**TABLE 1:** Sample preparation parameters for 3D printing of PLA/PHA/TPU blends.

Parameters	Unit	Levels		
Raster angle	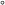	0	45/−45	90
Layer height	mm	0.3	0.4	0.5
Nozzle temperature	°C	200	210	220

**TABLE 2:** Design of experiment(L9).

S. No	Layer height (mm)	Nozzle temperature (°C)	Raster angle (°)
1	0.3	200	0
2	0.3	210	45/−45
3	0.3	220	90
4	0.4	200	45/−45
5	0.4	210	90
6	0.4	220	0
7	0.5	200	90
8	0.5	210	0
9	0.5	220	45/−45

### 2.3 Characterizations

#### 2.3.1 Scanning Electron Microscopy (SEM)

The cross‐sectional morphology of the PLA/APHA/TPU composite was examined using an electron microscope (INSPECT FE50) operating at 20 kV. Observations were conducted perpendicular to fractured surfaces on samples. The specimens were coated with a thin layer of gold through sputtering prior to investigation.

#### 2.3.2 Rheological Properties

The rheological properties of the PLA/APHA/TPU blend were evaluated using an Anton Paar rheometer (MCR 302, Germany) under dynamic oscillatory mode. The tests were conducted using a parallel plate geometry with a diameter of 25 mm. The experimental conditions included a 1 mm gap between the plates, a testing temperature of 210°C, a strain amplitude of 0.1%, and a frequency sweep spanning from 0.1 Hz to 100 Hz.

#### 2.3.3 Thermogravimetric Analysis (TGA)

The thermal decomposition behavior of PLA/APHA/TPU composites was analyzed using TGA (Q600, TA Instruments). Standard alumina crucibles were filled with specimens and heated under nitrogen at a rate of 10°C per min from 25°C to 700°C.

#### 2.3.4 Differential Scanning Calorimetry (DSC)

A differential scanning calorimeter (Q600, TA Instruments) was used to evaluate the thermal properties of PLA/PHA/TPU blends with sample weights ranging from 8 to 10 mg. Initially, the specimens were heated to 200°C at a rate of 10°C/min and held at this temperature for 5 min to remove any thermal history. They were then cooled to 25°C at 10°C/min. In the following steps, the specimens were heated at the same rate from 25°C to 200°C. The characteristic temperatures and enthalpies of the specimens were determined using a secondary melting curve. Thermal properties such as the glass transition temperature (T_g_), the cold crystallization temperature (T_cc_), and the melting temperature (T_m_) were measured. For the calculation of crystallinity (X_c_), the following equation has been used.

(1) $$X_{c}=\frac{\Delta H_{m}}{w_{f}\times \Delta H^{o}}\times 100$$

In this equation, $\Delta H_{m}$ represents the enthalpy of the polymer at melting, $w_{f}$ represents the polymer weight section in the blend or composite, and $\Delta H^{o}$ represents the enthalpy of melting of a theoretical 100% crystalline polymer. A DSC analysis can determine the % of crystallinity as, crystallization, glass transition temperature, and melting temperature of the polymer blends and composite.

#### 2.3.5 Mechanical Testing

The samples were left at room temperature for 48 h after the 3D printing process was completed. An Instron universal testing machine conducted uniaxial tensile tests (following ASTMD638) and three‐point flexural testing at a strain rate of 12 mm/min for ASTMD790. Using Static System, Inc.'s Izod machine, the impact properties of the PLA/APHA/TPU blend were determined according to ASTM D256. Three samples from each condition were used to calculate the standard deviation.

#### 2.3.6 Dynamic Mechanical Analysis (DMA)

The dynamic mechanical properties of the PLA/APHA/TPU blend were examined using a TA Instruments DMA Q800. Testing was conducted on rectangular specimens with dimensions of 35 × 5 × 2 mm^3^, utilizing the single cantilever mode for analysis. The testing temperature ranged from −70°C to 130°C, with a controlled heating rate of 3°C per minute and a test frequency of 1 Hz.

### 2.4 Heat and Force Responsive Shape Memory Property

PLA/APHA/TPU blends were evaluated for their force and heat shape recovery capabilities under bending loading modes under a controlled temperature environment through PID control. Figure S1 illustrates the schematics for a temperature control circuit with heaters, fans, and a PID controller. An integrated temperature‐controlled chamber has been installed to prevent heat loss from the UTM and apply heat and force simultaneously. A 3D‐printed rectangular specimen, measuring 100 × 12.7 × 1.5 mm^3^, was used for the Shape memory effect (SME) tests. Figure S2 illustrates the test cycle's representation and experimental shape recovery procedure.

In its original shape (rectangular), the 3D‐printed part was heated in a temperature‐controlled chamber by a PID controller to 39.5°C. Once the equilibrium condition is reached, the force is applied by bending test at the 5 mm/min strain rate until the deformation is 5 mm. An external force was applied until the specimen reached a hardened state after being cooled to room temperature. Angles are indicated by an α. To reheat the specimen, the heating was turned on while the sample was in the chamber. The chamber temperature remained at 39.5°C, and the sample was folded before being restored to its initial shape. Following the experimental process, the sample angle did not fully recover, as indicated by beta. To ensure accuracy, shape recovery tests were conducted three times for each sample. The equations below are used to determine the Shape Fixation Ratio (Rf) and Shape Recovery Ratio (Rr):

(2) $$R_{f}\left(\%\right)=\frac{\theta_{deformed}-\alpha }{\theta_{deformed\;}}*100$$

(3) $$R_{r}\left(\%\right)=\frac{\theta_{deformed}-\beta }{\theta_{deformed\;}}*100$$

A $\theta_{deformed}$ is the angle after a force is applied, a α is the fixed angle after removing the force and cooling it down, and a β represents the angle that remains unrecovered after the stimulus is applied.

#### 2.4.1 Multi‐Stimuli Heat and Force Response

In multi‐stimuli heat and force response, the sample is initially deformed at room temperature, followed by heating the chamber while maintaining force until the chamber reaches an equilibrium of 39.5°C shown in Figure S3. After removing the force, the sample is cooled down to room temperature. A sample is reheated to 39.5°C and observed to recover. Equations (1) and (2) were used to measure a shape fixity and shape recovery ratio.

#### 2.4.2 Sequential Deformation and Heating Response

Sequential heat and force responses are characterized by sequential application of external stimuli. Initially, the sample was deformed to a predetermined strain and then stored for a specific period to acquire its fixed shape. After removing the force, the chamber is heated to 39.5°C to observe the recovery. After the recovery is observed, the chamber is cooled down to room temperature shown in Figure S4.

#### 2.4.3 Dynamic Loading and Thermal Cycling

To evaluate the shape memory behavior under multi‐stimuli conditions, a thermal cycling and dynamic loading process was implemented. The testing chamber was heated to 39.5°C, and samples were held at this temperature to achieve thermal equilibrium. Shape recovery and fixity tests were performed over 10, 20, and 50 cycles, where force was cyclically applied and removed to induce and evaluate mechanical programming effects. Each cycle involved force application, removal, and reprogramming, as illustrated in Figure S5. After completing the required number of cycles, the samples were cooled to room temperature and subsequently reheated to assess their ability to return to their original shape.

## 3 Results and Discussion

### 3.1 Rheological Properties

**FIGURE 2:**
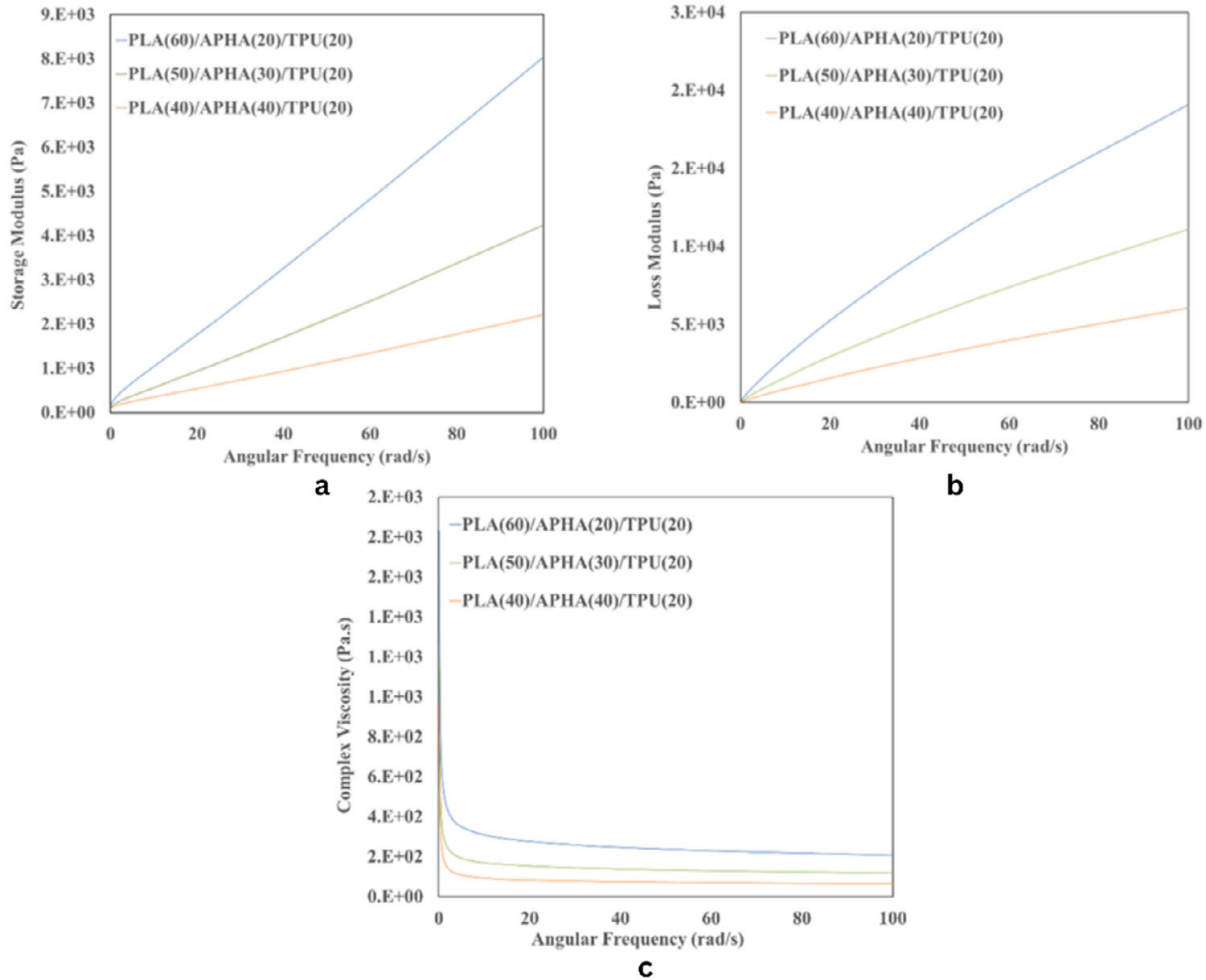
Variation of angular frequency with (a) storage modulus, (b) loss modulus, and (c) complex viscosity.

The rheological properties of the PLA/APHA/TPU blend are governed by the interactions of its polymer constituents within the matrix. Figure 2 illustrates the dependency of storage modulus (G), loss modulus (G″), and complex viscosity (η) on angular frequency, providing insights into the viscoelastic behavior and flow characteristics of the blend. The results indicate that both the storage modulus (G′) and loss modulus (G″) increase by increasing angular frequency, demonstrating the frequency‐dependent viscoelastic behavior of the material. While G″ remains higher than G′ across most of the test range, indicating a dominant viscous nature, there are specific points where G′ surpasses G″, suggesting a transition in the material's response. This variation highlights the complex interplay between elasticity and viscosity within the blend. The incorporation of APHA into the PLA/APHA/TPU blend led to a noticeable reduction in both the storage modulus (G′) and loss modulus (G″), indicating a decrease in stiffness and energy dissipation capability. This reduction is primarily due to the disruption of the polymer matrix by APHA, which weakens intermolecular interactions and reduces resistance to deformation. Likewise, as depicted in Figure 2(c), the complex viscosity (η) also decreases with increasing APHA content. This decrease is attributed to reduced molecular entanglement and weaker polymer interactions, which enhance the macroscopic fluidity of the blend. Furthermore, all samples demonstrated shear‐thinning behavior when subjected to increased angular frequencies, a phenomenon resulting from polymer chain disentanglement and realignment under shear stress. This confirms that the addition of APHA alters the blend rheological behavior, making it less resistant to deformation and flow.

### 3.2 Dynamic Mechanical Analysis

**FIGURE 3:**
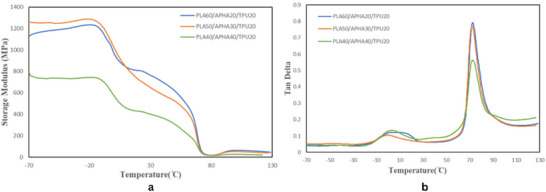
Dynamic mechanical analysis of the P/A/T blend with varying polymer compositions: (a) storage modulus G′ and (b) tan δ as functions of temperature.

DMA reveals the temperature‐dependent behavior of the PLA/APHA/TPU blends, as shown by the storage modulus (G′) and tan δ curves in Figure 3. As shown in Figure 3a, the storage modulus (G′) of the blend exhibits two distinct transitions. The first drop in G′ occurs around −10°C to 20°C (attributed to the glass transitions of APHA and TPU), and the second drop between ∼55°C and 80°C corresponds to the glass transition of PLA. Similarly, the tan δ plot (Figure 3b) reveals two glass transition peaks: a lower‐temperature peak around −4°C to 2°C from the APHA/TPU phases, and a higher peak around 72°C associated with the PLA phase. As the blend passes through each Tg, the storage modulus (E′) drops sharply due to increased chain mobility, reflecting the material's ability to undergo reversible deformation—a hallmark of SMPs [44]. Accordingly, 39.5°C was selected as the switching temperature (Ts) for shape recovery, since it is above the Tg of APHA and TPU (making those phases flexible) but still below PLA's Tg. In practice, when the deformed material is cooled below Ts (with PLA remaining glassy), PLA's rigid domains lock in the temporary shape, and upon reheating to Ts, the softened APHA and TPU phases enable the material to return to its original shape.

### 3.3 Thermal Behavior

**FIGURE 4:**
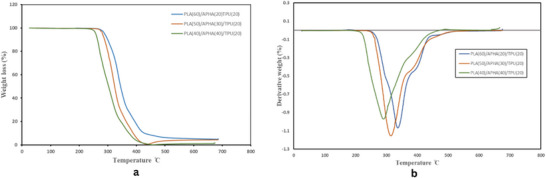
Thermal stability of the blend with different polymer compositions: (a) TGA profiles, (b) DTG profiles.

Figure 4 represents the TGA curves for PLA/APHA/TPU blends, with the corresponding decomposition parameters described in Table 3. For these samples, the initial degradation temperature for P60/A20/T20 occurred at 285°C, then reduced to 281.9°C for P50/A30/T20, and further declined to 273.9°C for P40/A40/T20. Also, the temperature corresponding to the maximum rate of weight loss (T_d, max_) decreased from 337.64°C to 287.73°C. This phenomenon can be attributed to the existence of APHA particles, which may reduce the overall thermal stability of the PLA/TPU/APHA composite. Due to its relatively lower degradation temperature, APHA can initiate earlier breakdown within the matrix, potentially facilitating the thermolysis process by weakening the PLA/TPU structure at elevated temperatures.

**TABLE 3:** Thermal parameters extracted from TGA data and DSC curves from the second heating cycle.

Specimen	T_d,5 wt.%_ (°C)	T_d, max _(°C)	T_g _(°C)	T_cc_ (°C)	T_m_ (°C)	ΔH_cc_ (J/g)	ΔH_m _(J/g)	X_C_(%)
P(60)/A(20)/T(20)	285.49	337.64	54.66	100.31	177.23	17.68	14.37	17.11
P(50)/A(30)/T(20)	281.86	315.66	52.53	95.15	175.52	14.34	13.80	19.71
P(40)/A(40)/T(20)	273.89	287.73	52.17	92.59	174.08	8.995	11.05	19.73

**FIGURE 5:**
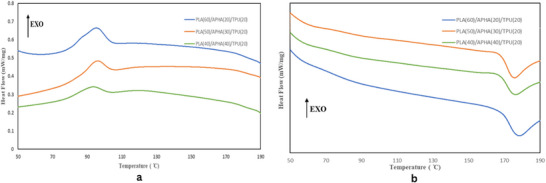
DSC curve of PLA blend with APHA and TPU (a) cooling curve and (b) second heating curve.

The melting and crystallization characteristics of the PLA/TPU/APHA blend were analyzed via DSC. The cooling and second heating curves, conducted after removing thermal history, are displayed in Figure 5. Key thermal parameters, including Tg, Tcc, Tm, ΔHcc, ΔHm, and Xc, are listed in Table 3. The addition of APHA and TPU to PLA, known for its inherently low crystallization rate [45], resulted in a distinct melt crystallization peak observed between 92°C and 100°C, as depicted in Figure 5a. In Figure 5b, Tg at 54.66°C and Tm at 177.23°C were observed for the P60/A20/T20 blend. Increasing the APHA content to 40% decreased Tg to 52.17°C and Tm to 174.08°C, likely due to enhanced chain mobility facilitated by APHA, despite the increase in overall crystallinity. Similarly, increasing the APHA content caused the cold crystallization peak (Tcc) to shift to a lower temperature, decreasing from 100.31°C for P60/A20/T20 to approximately 92°C for P40/A40/T20. This shift can be attributed to the increased crystallinity, as the higher APHA content promotes the formation of lamellar crystals during the cooling process, reducing the energy barrier for crystallization and enabling it to occur at a lower temperature. As calculated using Equation (1), the crystallinity (Xc, PLA) increased from 17.11% to 19.73% when the blend contained 40 wt.% APHA. This increase in crystallinity is significant, as PLA generally has low melt crystallization due to its stiff molecular structure. The presence of APHA likely facilitated improved chain mobility, allowing a greater proportion of PLA chains to reorganize and crystallize during the heating cycle.

### 3.4 Mechanical Properties and Toughness Enhancement

**FIGURE 6:**
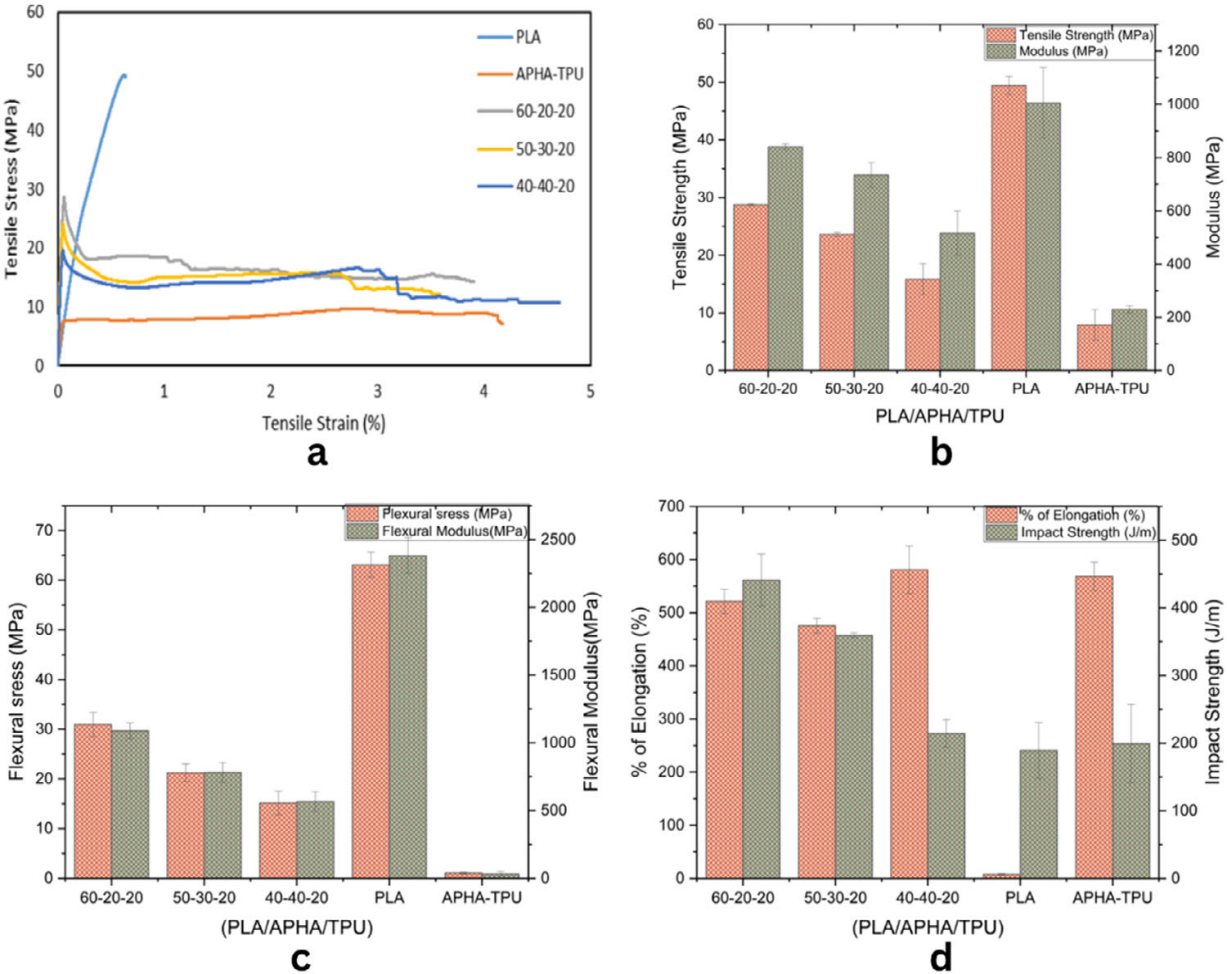
Mechanical performance of the specimens (a) stress‐strain graph, (b) tensile strength and modulus, (c) flexural stress and modulus, and (d) % of elongation and impact strength.

The impact of TPU and APHA on PLA's mechanical properties enhanced PLA's inherent brittleness. The tensile stress‐strain curves, as well as bar charts depicting the tensile strength, Young's modulus, Flexural stress, modulus, % of elongation, and impact strength, are presented in Figure 6. For the PLA/APHA/TPU blend with a composition of 60‐20‐20 wt.%, the tensile strength was measured at 28.79 ± 0.16 MPa. While this value is somewhat lower than the tensile strength of neat PLA, which is 49.42 ± 0.06 MPa, it reflects a significant enhancement over the individual strengths of APHA and TPU, recorded at 7.90 ± 2.66 MPa. This indicates that the blend effectively balances the mechanical properties of the constituent materials. The tensile strain reached approximately 5%, demonstrating that the incorporation of 20 wt.% TPU and 20 wt.% APHA Significantly improved the toughness (elongation at break) of the matrix while maintaining a well‐balanced mechanical strength. This is a significant improvement compared to neat PLA, which exhibits a tensile strain of around 0.6%. Compared to the PLA/APHA/TPU blend with a 60‐20‐20 composition, the tensile strength decreased slightly as the APHA content increased to 30 wt.%. Specifically, the tensile strength of the P50/A30/T20 blend is marginally lower than that of the P60/A20/T20 blend, measuring 23.62 MPa, while the P40/A40/T20 blend exhibits a further reduction to 15.87 MPa. The modulus of the PLA/APHA/TPU blend with a 60‐20‐20 composition slightly decreased to 840.17 MPa compared to neat PLA, which has a modulus of 1006.00 MPa. Also, as the APHA content increased, the modulus further declined to 735.36 MPa with the addition of 30 wt.% APHA and dropped to 516.34 MPa with 40 wt.% APHA. The flexural strength and modulus of the blend have decreased compared to neat PLA. Specifically, the flexural strength reduced from 63.12 MPa to 30.95 MPa, and the flexural modulus decreased from 2381.66 MPa to 1088.93 MPa in the P60/A20/T20 blend. However, when compared to the TPU/APHA 50/50 wt.% blend, there was a significant improvement, with a flexural strength of 1.02 MPa and a modulus of 32.50 MPa. This indicates that while the P60/A20/T20 blend shows lower mechanical properties than neat PLA, it performs better than the TPU/APHA blend in these aspects. Additionally, the elongation at break significantly increased to 521.26% in the P60/A20/T20 blend, compared to just 8.01% for PLA alone. This demonstrates a significant enhancement in toughness resulting from the incorporation of TPU and APHA into the PLA matrix. However, the tensile properties exceeded those reported in earlier studies, even when higher amounts of APHA and TPU were used. Ali et al. [46] reported that incorporating 25 wt.% of APHA into the PLA matrix increased the elongation at break to 183%. Ecker et al. [47] blended PLA with PHA and discovered that the addition of PHA nearly doubled the impact strength of the PLA, increasing it from 6.5 kJ/m^2^ to 12.7 kJ/m^2^.

### 3.5 Evaluation of 3D Printability and Morphological Analysis

In 3D printing, achieving strong adhesion between layers is essential for enhancing the mechanical strength of the final product [48]. In FDM printing, this interlayer bonding is primarily driven by the residual heat in the partially molten material, which promotes inter‐adhesion between successive layers [49]. Beyond this thermal effect, printing parameters critically influence the quality of layer bonding and can contribute to the formation of microvoids that affect the mechanical properties. PLA/APHA‐/PU blends were printed using different printing parameters and optimized to address these factors, ensuring consistent quality and improved mechanical properties throughout the layers.

**FIGURE 7:**
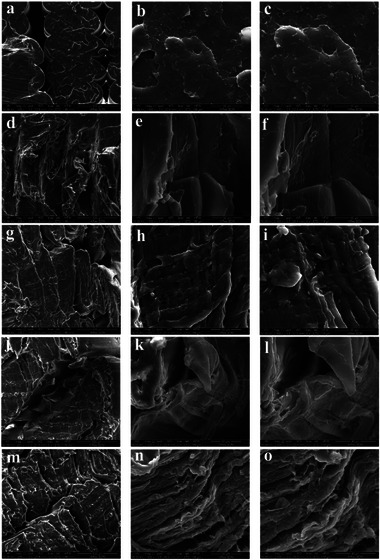
SEM analysis of PLA‐APHA‐TPU blends at different ratios and magnifications: (a–c) PLA (d–f) APHA‐TPU (g–i) 40/40/20 (j–l) 50/30/20 (m–o) 60/20/20.

The cross‐sectional morphologies of the fractured specimen were examined using SEM, as displayed in Figure 7a–o. Figure 7a–c shows the fractured surface of PLA, revealing characteristics of brittle fracture. This brittle behavior expected results from PLA's reduced ductility and low impact resistance, which lead to a sudden failure under stress. The fracture surface of the APHA/TPU composite, shown in Figure 7d–f, exhibits a sea‐island structure, indicative of a ductile fracture. This ductile behavior arises from TPU's inherent flexibility and impact resistance, which allow for energy absorption and plastic deformation prior to failure. The sea‐island morphology, with TPU as the dispersed phase, contributes to this enhanced toughness by effectively dissipating stress throughout the material [50]. While PLA/APHA and TPU exhibit shape memory properties, the shape memory effects of TPU and APHA were restricted due to their roles as dispersed phases within the blend. At this composition, TPU and APHA primarily served to enhance the toughness and increase the ductility of PLA, as demonstrated in the subsequent mechanical property evaluations [51].

In the PLA/APHA/TPU blends, SEM images of the cross‐sectional surface showed a uniform dispersion of TPU and APHA particles within the PLA matrix at TPU and APHA contents of up to 20 wt.% and 40 wt.% respectively, with no significant agglomeration observed. However, as APHA concentration increased, particle clustering became evident (Figure 7(g–i), suggesting that higher APHA loadings present challenges for maintaining uniform dispersion. This clustering is likely due to the limited compatibility and natural tendency of APHA and TPU to aggregate within the PLA matrix at elevated concentrations. Achieving a well‐dispersed distribution of APHA particles is thus crucial for optimizing the mechanical properties and shape‐memory response of the 3D‐printed composites, as particle agglomeration can weaken the material structure and reduce performance consistency. Figure 7(j–l) illustrates a more uniform dispersion of APHA and TPU within the matrix, contributing to improved mechanical and shape memory properties, as demonstrated in the following subsections.

### 3.6 Shape Memory Performance at Low Activation Temperature

**FIGURE 8:**
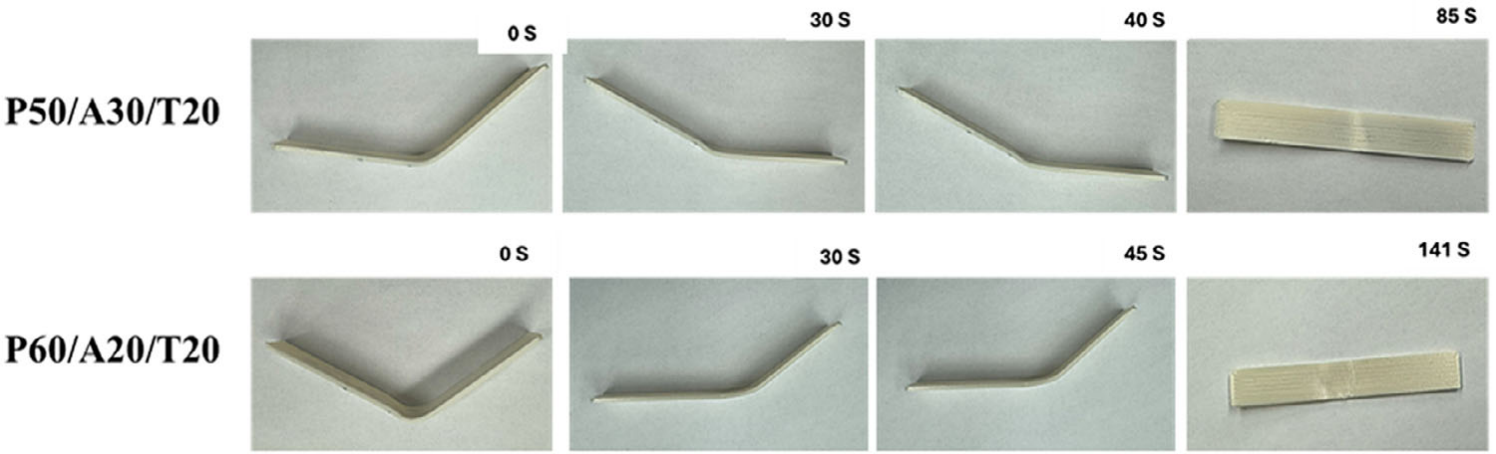
Shape memory behavior of PLA/APHA/TPU blend generated by force and heat.

A SMP operates effectively when it exhibits shape memory behavior, which is highly correlated with temperature and recovery time. In this study, the effect of heat and force on the shape memory behavior of PLA/APHA/TPU specimens was investigated as a function of time. The T_g_ for both APHA and TPU is very low; therefore, the shape responsive to heat could be quickly transmitted through the blend matrix. The UTM machine was equipped with a Heat Through Controlled Chamber, which was essential for transferring heat throughout the specimen and generating heat inside the polymer. Figure 8 illustrates the shape recovery process as well as instantaneous shapes.

All specimens were fabricated using 3D printing in a rectangular form with dimensions of 100 × 12.7 × 1.5 mm^3^. Temperature‐controlled chambers were used to achieve equilibrium before applying force. Once the temporary shape had been completed, the force was removed, and the sample was cooled to room temperature. The sample is placed again in the chamber for reheating after cooling as shown in the figure below. All PLA/APHA/TPU polymer blend specimens were placed in the same position. The original rectangular shape of a specimen returns when re‐heated. However, recovery times for specimens with different percentages varied. For example, P50/A30/T20 recovered their original shape after 85 s, while P60/A20/T20 recovered their original shape after 141 s at 39.5°C. Adding more than 20% of APHA reduced mechanical and shape recovery. The optimal material composition, P50/A30/T20, was found to enhance the shape recovery process. The entire procedure is documented and presented as Videos S1 and S2. These findings highlight the rapid shape response of the manufactured polymer blend at lower temperatures.

**FIGURE 9:**
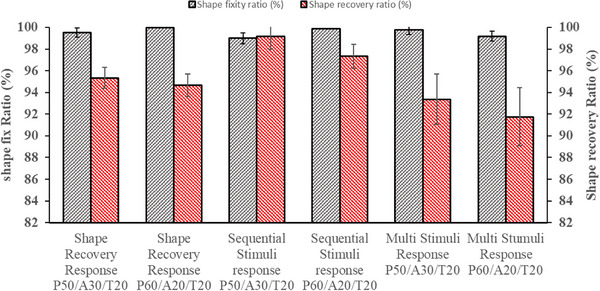
Shape memory performance of PLA/APHA/TPU blend: shape fixity ratio and shape recovery ratio.

**FIGURE 10:**
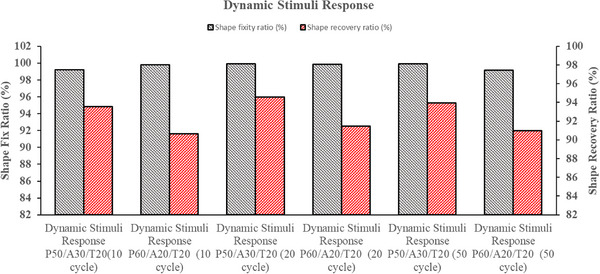
Shape memory performance of PLA/APHA/TPU blend under dynamic loading: shape fixity ratio and shape recovery ratio.

The shape‐fixity (Rf) and shape‐recovery (Rr) ratios were analytically evaluated under various conditions, including shape recovery, sequential, multi‐stimuli, and dynamic stimuli responses, as illustrated in Figures 9 and 10. All specimens exhibited exceptional shape fixity (Rf), ranging from 99% to 100% across various stimulus conditions. This behavior suggests that the relatively high modulus of rigid PLA effectively resists retraction, effectively counteracting the influence of APHA and TPU at ambient temperature [29].

In contrast, the shape recovery (Rr) values were recorded as 95.34%, 94.67%, 99.16%, 97.32%, 93.36%, and for the following responses, respectively: Shape Recovery Response P50/A30/T20, Shape Recovery Response P60/A20/T20, Sequential Stimuli Response P50/A30/T20, Sequential Stimuli Response P60/A20/T20, Multi‐Stimuli Response P50/A30/T20, and Multi‐Stimuli Response P60/A20/T20. During dynamic loading, shape recovery was successfully achieved across multiple cycles, with the following recovery (Rr) values: 93.58% for Dynamic Stimuli Response P50/A30/T20 (10 cycles), 90.64% for P60/A20/T20 (10 cycles), 94.56% for P50/A30/T20 (20 cycles), 91.46% for P60/A20/T20 (20 cycles), 93.96% for P50/A30/T20 (50 cycles), and 90.98% for P60/A20/T20 (50 cycles). Consequently, the PLA/APHA/TPU blends exhibited excellent shape‐fixity and recovery ratios, demonstrating their superior performance in maintaining and regaining shape under various conditions.

**FIGURE 11:**
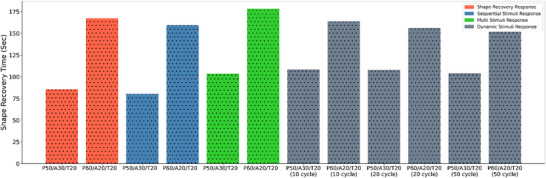
Shape memory performance of PLA/APHA/TPU blend: shape recovery time under various stimuli.

Figure 11 illustrates the shape recovery performance of each blend under different stimuli conditions, emphasizing the variations in recovery time. The comparison distinctly reveals each blend's unique responsiveness and efficiency, underscoring the impact of the applied stimuli on recovery dynamics. A clear trend emerges for the P50/A30/T20 blend, which consistently demonstrates a shape recovery time of approximately 85 s across all Stimuli that is Shape recovery response, multi‐stimulant, and sequential stimuli conditions. The dynamic loading stimulus results in a notably longer recovery time for the P50/A30/T20 blend, reaching approximately 110 s,—significantly higher than under other stimulus conditions. In contrast, the P60/A20/T20 blend exhibits a nearly identical recovery time of 160 s across all stimuli conditions; however, its recovery time is considerably longer compared to the P50/A30/T20 blend. Along with lowering the transition temperature and elastic modulus, increasing TPU and APHA concentrations of 20 wt.% and 30 wt.% respectively shortened the recovery time by reducing hard points and molecular entanglement within the PLA segments. In summary, reducing the PLA volume—which has a higher transition temperature and more rigid points—in PLA/APHA/TPU blends lowers the energy needed for shape recovery. This enables sufficient recovery energy to be achieved at lower temperatures. Consequently, increasing APHA/TPU content of 20 wt.% and 30 wt.% respectively correlate with a shorter shape recovery time, enhancing the recovery rate. This pattern of shape recovery variation has previously been observed in PLA blend samples [52, 53].

**FIGURE 12:**
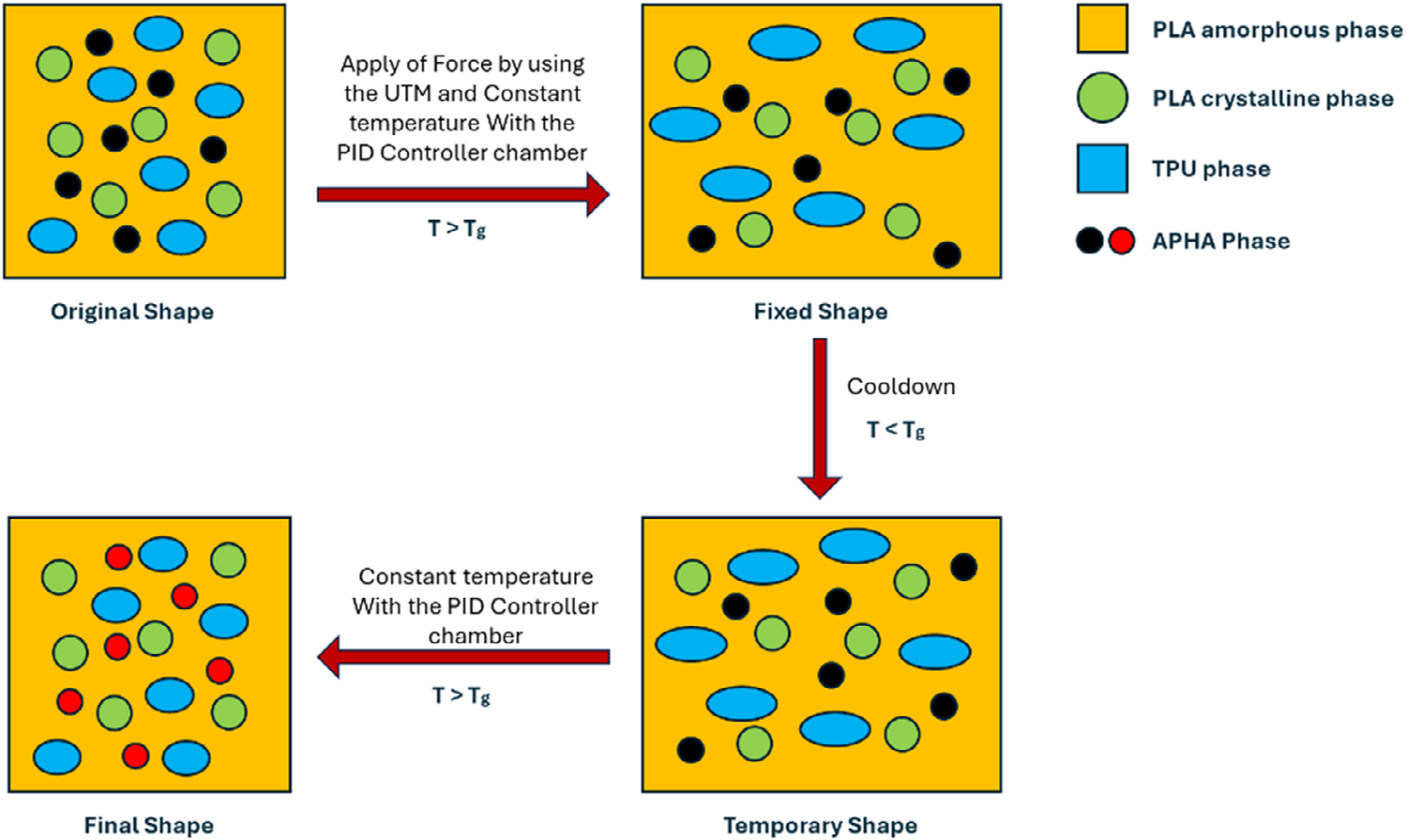
Force and heat‐induced shape memory effect mechanism in polymer blends.

As shown in Figure 12, the shape memory behavior of PLA/APHA/TPU composites in 4D printing utilizes the distinct functions of each component to enable efficient and adaptive shape recovery. PLA, being a semi‐crystalline polymer, contains crystalline regions that function as the fixing phase, maintaining the original shape, while its amorphous regions serve as the switching phase, allowing for temporary shape formation [51]. This dual‐phase structure underpins PLA's role as a widely applied SMP with inherent self‐recovery capabilities [54].

Incorporating small amounts of TPU significantly improves the composite's toughness and ductility, allowing it to withstand deformation and recover without permanent damage, especially at ambient temperatures. Additionally, APHA enhances the composite's flexibility and responsiveness, enabling tailored shape recovery under a variety of stimuli. Together, these interactions between PLA, TPU, and APHA create a balanced, tunable shape memory behavior, advancing the applicability of PLA/APHA/TPU composites for complex, multi‐functional applications in 4D printing.

In the PLA/APHA/TPU composite blend, the shape memory mechanism is activated even below the glass transition temperature (Tg), specifically at 39.5°C, demonstrating effective shape recovery at a relatively mild thermal input. This recovery response, achieved at a temperature lower than the Tg, is likely attributed to the synergetic interaction among PLA, APHA, and TPU components within the blend. The elastic energy stored in the matrix is gradually released at 39.5°C, allowing the material to revert to its original configuration under these milder thermal conditions. The TPU component provides the composite with the necessary ductility and resilience, safeguarding against permanent deformation during the shape recovery process. Meanwhile, APHA enhances flexibility and thermal responsiveness, facilitating a reliable, low‐temperature recovery. This finely tuned, low‐temperature shape memory behavior underscores the PLA/APHA/TPU composite's potential for advanced 4D printing applications such as orthopedic braces, where precise shape recovery under controlled, lower‐temperature stimuli is crucial for durability and adaptability.

## 4 Conclusions

This study introduces a novel PLA/APHA/TPU composite for 4D printing that successfully combines high mechanical strength with low‐temperature shape recovery, effectively overcoming the key limitations associated with conventional PLA‐based SMPs. The optimized formulation exhibits superior mechanical properties and consistent actuation behavior, rendering it highly suitable for biomedical and adaptive applications. Notably, the composite achieves rapid shape recovery at approximately 39.5°C, significantly lower than the glass transition temperature of neat PLA (∼60°C), and demonstrates nearly 100% shape fixity with up to 99% recovery under various thermal and mechanical programming conditions. These features ensure reliable and repeatable actuation in real‐world environments.

The incorporation of thermoplastic PU (TPU) enhances toughness and elasticity, reducing the risk of permanent deformation during repeated loading cycles, while the addition of amorphous polyhydroxyalkanoate (APHA) contributes to improved flexibility and a lowered activation temperature. This synergistic combination delivers a distinctive balance of strength, ductility, and responsiveness at lower temperatures, surpassing the performance of traditional SMP systems. The 60/20/20 wt.% PLA/APHA/TPU blend demonstrated markedly superior tensile strength, elongation, and modulus compared to both neat PLA and PLA/TPU composites, achieving a notable 500% increase in elongation at break. This impressive performance provides the resilience and toughness needed for load‐bearing applications without sacrificing flexibility.

The composite's low‐temperature activation, coupled with its enhanced mechanical robustness, makes it an ideal candidate for 4D‐printed applications such as orthopedic braces, soft robotic actuators, and adaptive medical implants. Its ability to undergo reliable shape transformations at body‐safe temperatures improves both user comfort and functional performance in patient‐specific devices. Furthermore, the use of biodegradable, bio‐based components—PLA and APHA—offers a sustainable and biocompatible alternative to petroleum‐based SMPs, contributing to environmentally responsible development in the biomedical sector.

## Conflicts of Interest

The authors declare no conflicts of interest.

## Declaration of Generative AI and AI‐Assisted Technologies in the Writing Process

During the preparation of this work, the author(s) used ChatGPT to improve language and readability. After using this tool/service, the author(s) reviewed and edited the content as needed and take(s) full responsibility for the content of the publication.

## Supporting information




**Supporting File 1**: marc70022‐sup‐0001‐SuppMat.docx.

**Supporting File 2**: marc70022‐sup‐0002‐VideoS1.mp4.

**Supporting File 3**: marc70022‐sup‐0003‐VideoS2.mp4.

## Data Availability

The data that support the findings of this study are available from the corresponding author upon reasonable request.
