# Supplementary material for: Dual‐Stimuli Responsive and Sustainable PLA/APHA/TPU Blend for 4D Printing
Source: Macromol Rapid Commun. 2025 Aug 14;46(21):e00414. doi: 10.1002/marc.202500414 (PMC12590943; doi:10.1002/marc.202500414)
Supplement: Supplementary file 1 — Supporting File 1: marc70022‐sup‐0001‐SuppMat.docx. [file MARC-46-e00414-s003.docx]

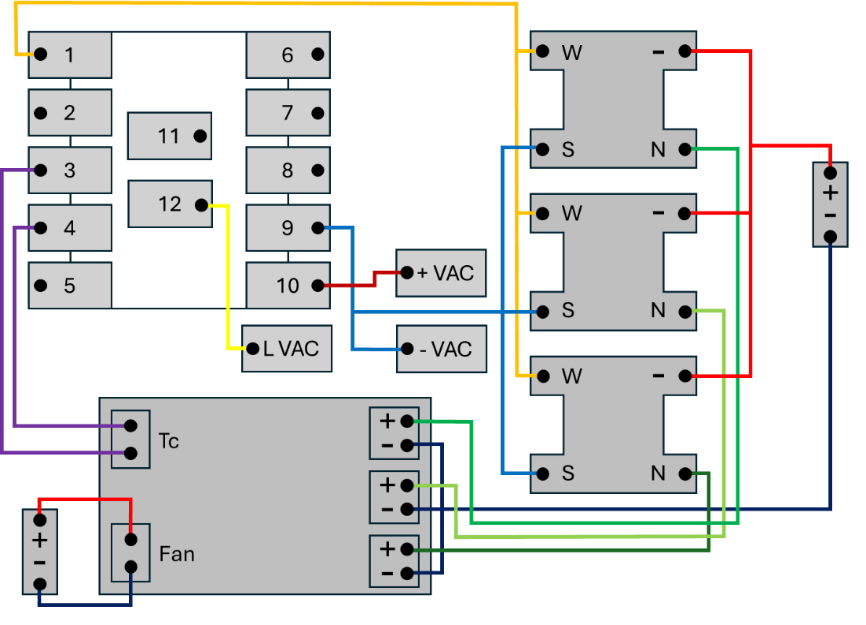


Figure 1S. Schematic diagram of the electrical circuit for the heating chamber


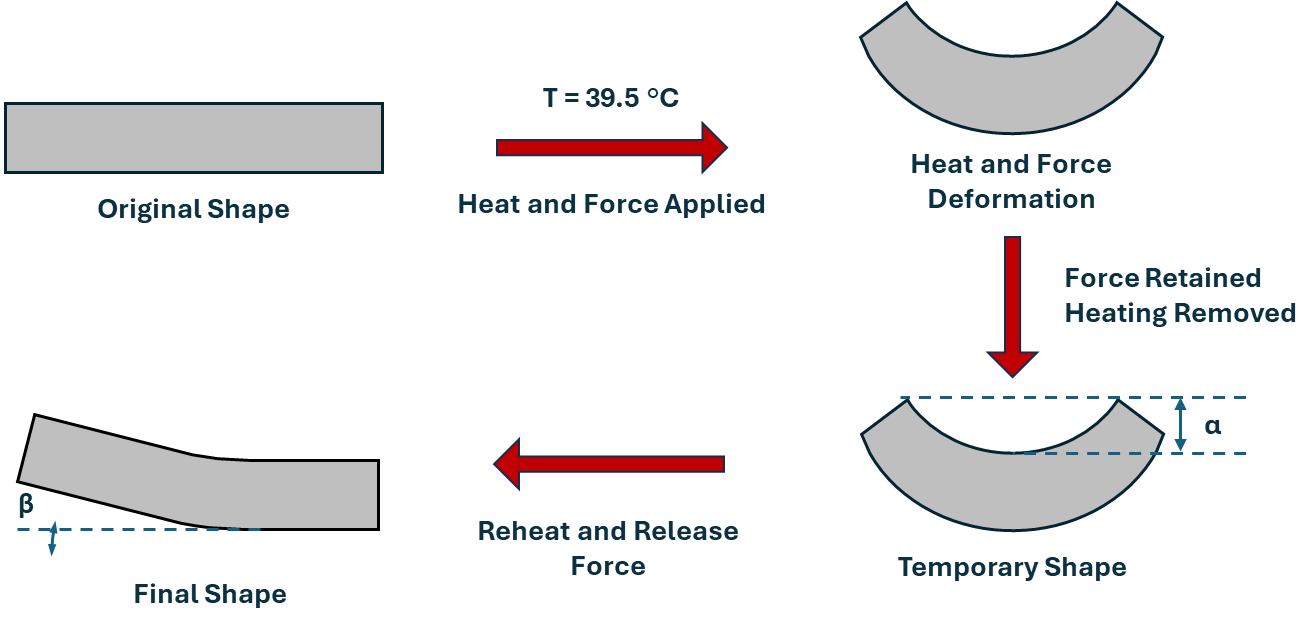


Figure 2S. Schematic representation of the shape memory response process


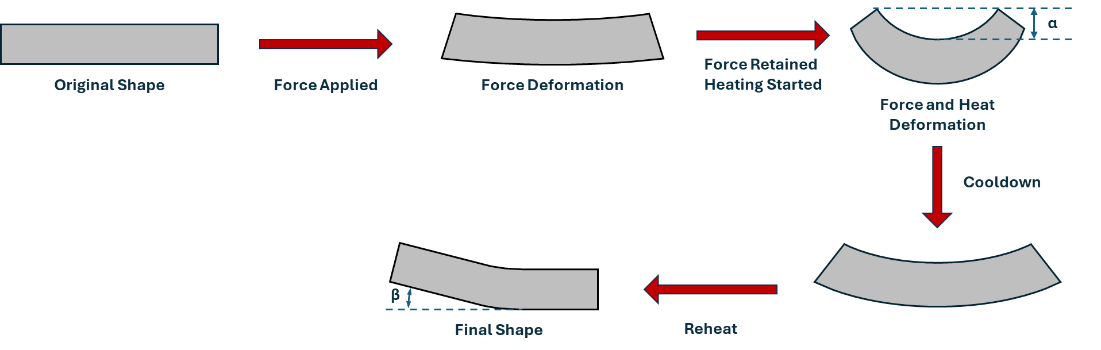


Figure 3S. Schematic representation of the Multi-Stimuli Heat and Force Response


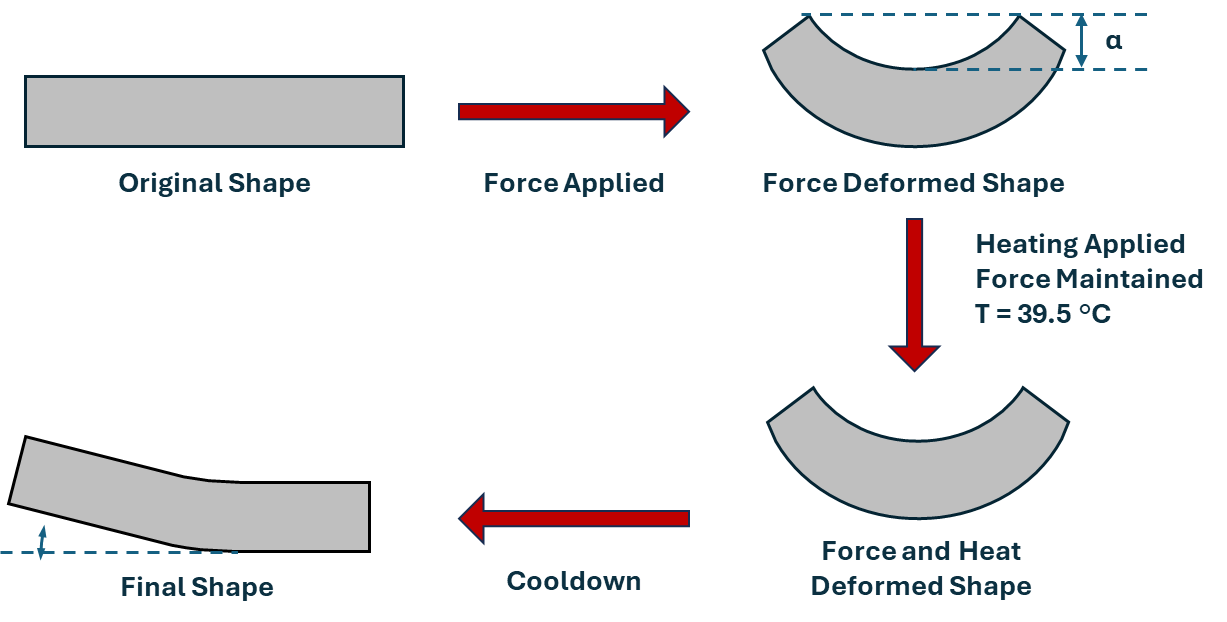


Figure 4S. Schematic representation of the Sequential Deformation and Heating Response


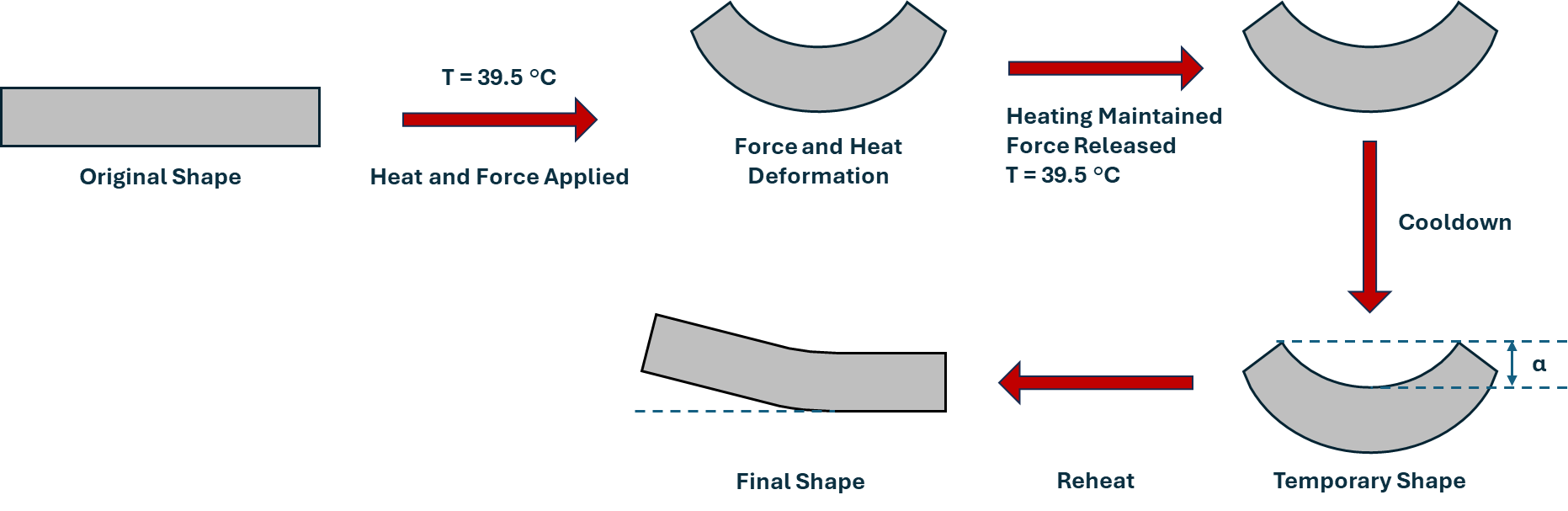


Figure 5S. Schematic representation of Dynamic loading and thermal cycling
